# King Edward's Hospital Fund for London

**Published:** 1920-01-10

**Authors:** 


### King Edward's Hospital Fund for London.

A MEETING of the General Council of King Edward's Hospital Fund for London was held on January 5 at the offices of the Fund, 7 Walbrook, E.C. 4. There were present: The Earl of Bessborough, in the Chair; the Lord Somerleyton (Honorary Secretary); Lord Stuart of Wortley, K.C.; Sir William Church, Bt.; Sir John Tweedy; and Mr. John G. Griffiths (Honorary Secretary). The Order, signed by His Royal Highness the Prince of Wales, President of the Fund, appointing Members of the General Council, Members of Committees, and Officers, for the year 1920, was read. The resolutions providing for the work of the Fund for 1920, which were approved at the meeting of the President and General Council held at St. James's Palace on December 16, 1919, were formally adopted.

